# Corrigendum to “A New Method of Scanning Tunneling Spectroscopy for Study of the Energy Structure of Semiconductors and Free Electron Gas in Metals”

**DOI:** 10.1155/2021/7626725

**Published:** 2021-01-21

**Authors:** A. Pavlov, H. Ihantola

**Affiliations:** Laboratory of Electronics and Information Technology, University of Turku, Turku, Finland

In the article titled “A New Method of Scanning Tunneling Spectroscopy for Study of the Energy Structure of Semiconductors and Free Electron Gas in Metals” [1], the authors identified an error in Equation (3), which should be corrected as follows:

Original text

(3) $$\begin{array}{c} I_{t}\propto \frac{1}{l}\left(E_{gn}\exp\left(\xi \frac{eV-{eV}_{SPV}}{E_{gn}}\right)-E_{gp}\exp\left(\xi \frac{-eV-{eV}_{SPV}}{E_{gp}}\right)\right) \end{array}$$

The corrected formula (3):

(3) $$\begin{array}{c} I_{t}\propto \frac{1}{l}\left(E_{gn}\exp\left(\xi \frac{eV-{eV}_{SPV}}{E_{gn}}\right)-E_{gp}\exp\left(\xi \frac{-\left(eV-{eV}_{SPV}\right)}{E_{gp}}\right)\right) \end{array}$$
